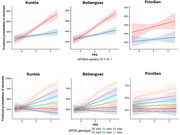# Non‐*APOE* Polygenic risk score derived from European ancestry data are more predictive of dementia among Hispanics who are *APOE* ε4 carriers

**DOI:** 10.1002/alz.095718

**Published:** 2025-01-09

**Authors:** Yuexuan Xu, Dolly Reyes‐Dumeyer, Tamil Iniyan Gunasekaran, Min N Qiao, Angel Piriz, Rafael A. Lantigua, Martin Medrano, Diones Rivera Mejia, Badri N. Vardarajan, Richard Mayeux

**Affiliations:** ^1^ Columbia University, New York, NY USA; ^2^ Gertrude H. Sergievsky Center, Vagelos College of Physicians & Surgeons, Columbia University, New York, NY USA; ^3^ The Taub Institute for Research on Alzheimer’s Disease and the Aging Brain, Vagelos College of Physicians & Surgeons, Columbia University, New York, NY USA; ^4^ The Gertrude H. Sergievsky Center, College of Physicians and Surgeons, Columbia University, New York, NY USA; ^5^ Taub Institute for Research on Alzheimer’s Disease and the Aging Brain, Columbia University, New York, NY USA; ^6^ G.H. Sergievsky Center, Columbia University, New York, NY USA; ^7^ Department of Medicine, Vagelos College of Physicians & Surgeons, Columbia University, New York, NY USA; ^8^ Pontificia Universidad Católica Madre y Maestra, Santiago, Santiago Dominican Republic; ^9^ CEDIMAT, Plaza de la Salud, Santo Domingo, Santo Domingo Dominican Republic; ^10^ Universidad Pedro Henríquez Urena, Santo Domingo Dominican Republic; ^11^ Taub Institute for Research on Alzheimer’s Disease and the Aging Brain, Vagelos College of Physicians and Surgeons, Columbia University, New York, NY USA; ^12^ Departments of Neurology, Psychiatry, and Epidemiology, Gertrude H. Sergievsky Center, The Taub Institute for Research on Alzheimer’s Disease and the Aging Brain, Vagelos College of Physicians and Surgeons, Columbia University, New York, NY USA

## Abstract

**Background:**

Despite concerns about the transferability of polygenic risk scores (PRS) derived from European ancestry data to Hispanics, recent research suggests that many genetic loci identified through European ancestry genome‐wide association studies (GWAS) for complex traits are also relevant in Hispanics. Furthermore, studies on dementia have shown improved PRS performance in this group, even with PRS developed from European GWAS. Recent research also indicates that *APOE* ε4‐independent PRS associations vary depending on *APOE* ε4 status. This study evaluates the relationship between multiple recent European GWAS‐derived *APOE*‐independent PRS in Hispanics and the interaction between *APOE* ε4 and PRS in relation to Alzheimer’s Disease (AD).

**Methods:**

We constructed a range of PRS based on three recently published GWAS (Kunkle et al., 2019, Bellenguez et al., 2022, FinnGen) of European ancestry among 1,429 Hispanics from the Washington Heights‐Hamilton Heights‐Inwood Community Aging Project (WHICAP) using clumping and thresholding methods. For each GWAS, we first evaluated the associations of several *P*‐value thresholds with AD to determine the threshold for further analysis. Empirical *P*‐values were used to avoid overfitting for the optimized PRS. We then tested whether and how *APOE* ε4 can modify the association between PRS and AD. All associations were fitted by logistic regression with sex, age, education, and the first 5 principal components as covariates.

**Results:**

Across all European GWAS, the optimal *P*‐threshold for the most predictive PRS is at a conservative *P*‐threshold (Kunkle: 2.87e‐6, Bellenguez: 1.11e‐6, FinnGen: 1.80e‐5). PRS constructed based on the Bellenguez GWAS are most strongly associated with AD (OR = 1.29, 95% CI: 1.17‐1.42), and the FinnGen‐derived PRS show the least association (OR = 1.06, 95% CI: 0.98‐1.14). The association of all three PRSs is stronger among *APOΕ* ε4 carriers (OR_Kunkle_: 1.51, 95% CI: 1.26‐1.81; OR_Bellenguez_: 1.60, 95% CI: 1.35‐1.91; OR_FinnGen_: 1.12, 95% CI: 0.97‐1.26) compared to non‐carriers (OR_Kunkle_: 1.09, 95% CI: 0.96‐1.23; OR_Bellenguez_: 1.16, 95% CI: 1.03‐1.31; OR_FinnGen_: 1.03, 95% CI: 0.93‐1.14). Similar findings were observed concerning cognition and progression to dementia.

**Conclusion:**

PRS derived from a European GWAS identified individuals at high risk for AD dementia among Hispanics, and the association is strongest among *APOE* ε4 carriers.